# SENP2 regulates MMP13 expression in a bladder cancer cell line through SUMOylation of TBL1/TBLR1

**DOI:** 10.1038/srep13996

**Published:** 2015-09-15

**Authors:** Mingyue Tan, Hua Gong, Jun Wang, Le Tao, Dongliang Xu, Erdun Bao, Zhihong Liu, Jianxin Qiu

**Affiliations:** 1 Department of Urology, Shanghai First People’s Hospital, Medical College of Shanghai Jiao Tong University, Shanghai 200080, China; 2 Shanghai Tianyou Hospital, Tongji University, Shanghai 200331, China

## Abstract

Bladder cancer (BC) is the most popular malignant urinary cancer in China. BC has the highest incidence and mortality among all genitourinary system tumors. Although the early-stage BC could be treated with advanced electron flexible systourethroscope, early metastasis of the BC occur frequently, and often results in poor prognosis. Recently, we reported that small ubiquitin related modifier (SUMO)-specific protease 2 (SENP2) was downregulated in BC specimen. SENP2 appeared to inhibit migration and invasion of bladder cancer cells *in vitro*, through suppressing MMP13 in BC cells. However, the exact underlying mechanisms remain unknown. Here, we reported that SENP2 inhibited nuclear translocation of β-catenin, which targeted the promotor of MMP13 to activate MMP13 to enhance BC cell metastasis. WNT ligands induced TBL1/TBLR1 SUMOylation to form complexes with β-catenin to facilitate β-catenin nuclear translocation, which could be efficiently inhibited through suppression of SUMOylation of TBL1/TBLR1. Together, our data suggest that SENP2 inhibits MMP13 expression in BC cells through de-SUMOylation of TBL1/TBLR1, which inhibits nuclear translocation of β-catenin. Thus, SENP2 may be a promising therapeutic target for BC.

The WNT-β-catenin signaling pathway is essential for cell differentiation and proliferation during development and homeostasis, and during tumorigenesis^1^,^2^,^3^,^4^. Aberrant activation of the WNT-β-catenin signaling pathway has been found to be associated with a variety of human cancers, including bladder cancer (BC)^5^,^6^,^7^,^8^,^9^. A key player of WNT-β-catenin signaling is the level of cytosolic β-catenin and nuclear β-catenin^1^,^2^,^3^,^4^. In response to WNT signals normally initiated by ligand bindings, β-catenin is accumulated in cytoplasm and transported to the nucleus, where it binds to the T cell factor (TCF)/lymphoid-enhancing factor (LEF) proteins^1^,^2^,^3^,^4^. There β-catenin reassembles the corepressor complexes containing HDACs to β-catenin/TCF/LEF-mediated transcriptional activation by recruitment of different coactivators^1^,^2^,^3^,^4^.

TBL1 and its related gene TBLR1 both contain a LisH/WD-40 motif^10^,^11^,^12^,^13^,^14^,^15^,^16^,^17^. Recent studies have shown that TBL1/TBLR1 complex targets NCoR/SMRT corepressor complexes, leading to the stable repression of target genes, such as deiodinase I, suggesting a role of TBL1/TBLR1 in class I HDAC corepressor complex-mediated transcriptional repression^10^,^11^,^12^,^13^,^14^,^15^,^16^,^17^. Moreover, TBL1 and TBLR1 were also found to selectively serve as mediators for the exchange of the nuclear receptor corepressors NCoR/SMRT upon ligand binding/stimulation^10^,^11^,^12^,^13^,^14^,^15^,^16^,^17^. Hence, TBL1/TBLR1 may functions as an E3 ubiquitin ligase adaptor for the recruitment of specific ubiquitin/proteasome machinery to degrade the corepressor for specific nuclear receptor-mediated gene activation^10^,^11^,^12^,^13^,^14^,^15^,^16^,^17^. Thus, these findings suggest a function of TBL1/TBLR1 as a transcriptional coactivator for WNT target genes^10^,^11^,^12^,^13^,^14^,^15^,^16^,^17^. Indeed, it has been recently reported that TBL1/TBLR1 is SUMOylated by WNT ligand bindings^10^.

SUMO is a small ubiquitin-like protein which is covalently attached to proteins through forming isopeptide bonds with specific lysine residues of target proteins^18^,^19^,^20^,^21^,^22^. The mammalian SUMO protein family includes 4 members (SUMO-1–4)^18^,^19^,^20^,^21^,^22^. SUMO targets lysine by an enzymatic cascade composed of 3 enzymes: E1 (Uba2/Aos1), E2 (Ubc9), and E3 ligases^18^,^19^,^20^,^21^,^22^. SUMO conjugation is initiated by formation of a thioester bond with the activating enzyme E1, a heterodimer of Aos1 and Uba2^18^,^19^,^20^,^21^,^22^. Afterwards, Aos1/Uba2 transfers SUMO to the single E2-conjugating enzyme Ubc9, which is responsible for SUMOylation of the substrate^18^,^19^,^20^,^21^,^22^. The Ubc9 substrate recognition is substantialized by specific E3 SUMO ligases^18^,^19^,^20^,^21^,^22^. SUMOylation is a dynamic process that is readily reversed by a family of SUMO-specific proteases, among which SUMO-specific protease 2 (SENP2) is a nuclear-envelope-associated protease^23^,^24^,^25^,^26^,^27^,^28^. The mouse SENP2 has been found to play a critical role in the embryonic cardiac development and the dynamics and functional maintenance of neuron system^23^,^24^,^25^,^26^,^27^,^28^.

Recently, we reported that SENP2 was downregulated in BC specimen. SENP2 appeared to inhibit bladder cancer cells migration and invasion *in vitro*, through suppressing MMP13 in BC cells^29^. However, the exact underlying mechanisms remain unknown.

Here, we reported that SENP2 inhibited nuclear translocation of β-catenin, which targeted the promotor of MMP13 to activate MMP13, which enhanced BC cell metastasis. WNT ligands induced TBL1/TBLR1 SUMOylation to form complexes with β-catenin to facilitate β-catenin nuclear translocation, which was efficiently inhibited through suppression of SUMOylation of TBL1/TBLR1. These data suggest that SENP2 inhibits MMP13 expression in BC cells through de-SUMOylation of TBL1/TBLR1, which inhibits nuclear translocation of β-catenin. SENP2 may be a promising therapeutic target for BC.

## Materials and Methods

All the experimental methods in the current study has been approved by the research committee at Medical College of Shanghai Jiao Tong University. All the experiments have been carried out in accordance with the guidelines from the research committee at Medical College of Shanghai Jiao Tong University.

### Cells and Reagents

Human BC cell line T24 cells were purchase from American Type Culture Collection (ATCC, Rockville, MD, USA) and cultured in RPMI 1640 medium supplemented with 10% heat-inactivated fetal bovine serum (FBS, Invitrogen, Carlsbad, CA, USA), 100 U/ml penicillin and 100 μg/ml streptomycin (Invitrogen) in a humidified atmosphere of 5% CO_2_ at 37 °C. Recombinant human WNT5a (used at 400 ng/ml) was purchased from R&D Systems (Minneapolis, MN, USA).

### Cytoplasmic and Nuclear Extracts

Cytoplasmic and nuclear fractions were prepared by using the NE-PER nuclear and cytoplasmic extraction kit (Life technologies, Grand Island, NY, USA) according to the manufacturer’s protocol. In brief, the cell pellet was re-suspended in ice-cold reagent and incubated on ice for 10 min. After incubation, the contents of the tube were mixed and centrifuged at 16,000 × g for 5 min, after which the supernatant was collected (cytoplasmic fraction). The insoluble fraction, which contains nuclei, was re-suspended in another reagent, incubated on ice for 40 min, centrifuged at 16,000 × g for 10 min, after which the supernatant (nuclear fraction) was collected. Each of these fractions was then analyzed for purity by immunoblotting for specific protein markers (for cytoplasmic fraction, α-tubulin; and for nuclear fraction, LaminB1). Samples containing 10 μg of total protein were separated by SDS-PAGE, transferred to nitrocellulose, and probed for specific proteins. Immunoreactive bands were detected using the ECL system. All immunoblotting experiments were repeated at least 4 times with similar results.

### Constructs and Mutagenesis

SENP2-overexpressing T24 cells have been prepared as has been described before^29^. Lysine 497 and 560 were mutated to arginine using the QuikChange XL Site-directed Mutagenesis Kit (Stratagene, La Jolla, CA, USA) and appropriate mutant primers. A double mutant was generated by subsequent mutagenesis. All mutations were confirmed by sequence analysis.

### *In Vitro* and *in Vivo* SUMOylation Assay

*In vitro* SUMOylation assays were performed using the SUMOylation kit (Enzo Life Sciences International, Inc., Plymouth Meeting, PA, USA) according to the manufacturer’s protocol. In brief, 200 nmol/l of purified recombinant human TBL1 or TBLR1 protein was incubated with reaction mixture containing 50 mmol/ Tris-HCl (pH 7.4), 2 mmol/l DTT, 5 mmol/l ATP, 10 mmol/l MgCl_2_, Aos-1 (150 ng), His-Uba2 (400 ng), GST-Ubc9 (500 ng), and GST-SUMO-1 for 1 hour at 30 °C. The control reaction was carried out in the absence of ATP. After the incubation, protein SUMOylation was identified by immunoblotting using the anti-SUMO antibody provided with the kit. For *in vivo* assays, T24 cells were treated with WNT5a for 30 minutes, after which the nuclear extracts were prepared as described above. Extracts (200 μg of protein) were immunoprecipitated with anti-SUMO-1 antibody (Santa Cruz Biotechnology, Dallas, Texas, USA) followed by immunoblotting with anti-TBL1 or anti-TBLR1.

### Chromatin Immunoprecipitation Assay (ChIP)

A ChIP assay was performed to assess *in vivo* DNA-protein interactions at the MMP13 promoter, using a ChIP assay kit (Upstate Biotechnology, Inc., Lake Placid, NY, USA) according to the manufacturer’s instructions. Briefly, 5 × 10^6^ cells were harvested and fixed in 1% (v/v) formaldehyde for 10 min at room temperature. After washing with cold phosphate-buffered saline, cells were lysed in 1% SDS for 30 min on ice. The lysates were sonicated to shear DNA using a Branson 250 sonicator (two 15-s pulses at 40% power in an ice bath, with 1 min between each pulse). These shearing conditions generate DNA fragments ranging in size from 500 to 1,000 bp. Chromatin solution was precleared with protein G-coated magnetic beads for 2 hours at 4 °C. Ten microliters of the chromatin solution was reserved as the “input” sample. The remaining chromatin was immunoprecipitated overnight at 4 °C with 3 μg of antibody specific to β-catenin, rabbit immunoglobulin G (IgG) as a negative control (provided with the kit). The chromatin-antibody complexes captured on the beads were washed several times and then eluted in 50 μl of elution buffer. The immunoprecipitated and input sample cross-links were reversed by incubation for 2.5 hours at 65 °C. After treatment with proteinase K at for 1 hour at 37 °C, the reaction was stopped and the resulting DNA stored at −20 °C until analyzed by standard and real-time quantitative PCR (RT-qPCR) as described below.

### Semi-quantitative PCR

PCR analysis was performed in a 50-μl volume containing 5 μl of ChIP DNA, 1 μmol/l of each primer, 2 mmol/l MgCl_2_, 0.2 μmol/l dNTP, and 0.04 units/μl of AmpliTaq Gold DNA polymerase. The PCR conditions were as follows: 1 cycle at 94 °C for 3 min, 35 cycles at 94, 60, and 72 °C for 30 seconds each, and a final cycle at 72 °C for 10 min. PCR products were analyzed by agarose gel electrophoresis. The primers used in the PCR were designed to amplify the promoter region of the human MMP13 gene (primer sets for MMP13 promoter, forward: 5′-GCCAGATGGGTTTTGAGAC-3′ and reverse: 5′-GTGATGCCTGGGGACTGTT-3′).

### RT-qPCR

Total RNA was isolated using an RNeasy Mini kit (Qiagen, Valencia, CA, USA). Two micrograms of total RNA was reverse transcribed for 1 hour at 42 °C using an avian myeloblastosis virus reverse transcriptase and oligo(dT) primer. Then 1 μl of reverse transcriptase solution was combined in a reaction mixture with 1 μl of the specific primer pair, 7.5 μl of 2X SYBR Green PCR Master Mix, and water to a final reaction volume of 15 μl. Primers were all purchased from Qiagen. RT-qPCR were then run in duplicate, with 40 cycles of amplification, on an ABI Prism 7000 real time PCR instrument (Applied Biosystems, Foster City, CA, USA). A negative control containing primers, water, and Master Mix, but no complementary DNA, was included. The relative gene transcripts were first normalized to the internal control TATA box-binding protein, calculated with the comparative cycle threshold (ΔΔCt) method, and then compared to experimental controls. Relative fold changes were presented.

### Statistical Analysis

Data were expressed as means ± standard deviation (SD). Statistical significance was evaluated by Student’s t test or one-way analysis of variance (ANOVA) followed by the Fisher’s Exact Test. Differences were considered statistically significant at p < 0.05.

## Results

### Preparation of SENP2-overexpressing BC cells

**Figure 1:**
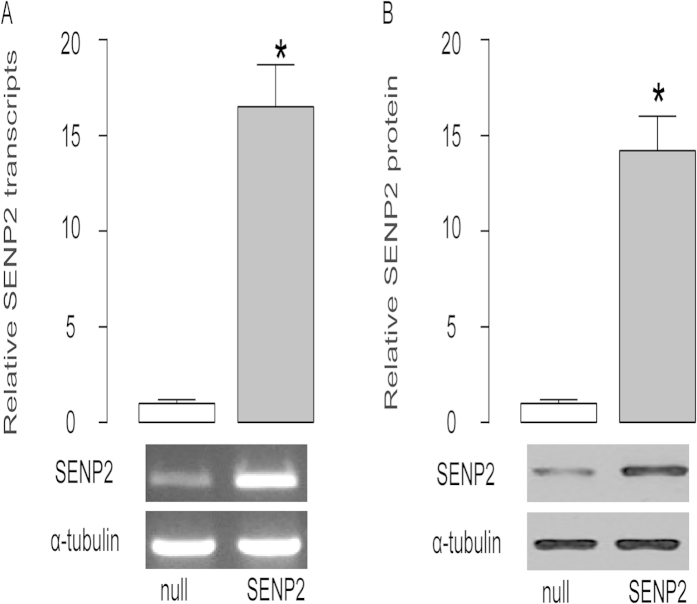
Preparation of SENP2-overexpressing BC cells. (**A**–**B**) In order to molecular mechanisms that regulate the effects of SENP2 on MMP13 and BC cell metastasis, we have prepared SENP2-overexpressing T24 cells. T24 cells that were transduced with a null construct were used as a control (null). The SENP2 levels in these cells were analyzed, by RT-qPCR (a representative gel from an accompanying semi-quantitative PCR was also shown) (**A**) and by Western blot (**B**). *p < 0.05. N = 5.

In order to molecular mechanisms that regulate the effects of SENP2 on MMP13 and BC cell metastasis, we have prepared SENP2-overexpressing T24 cells. T24 cells that were transduced with a null construct were used as a control (null). The SENP2 levels in these cells were analyzed, which confirmed the modification of SENP2 in T24-SENP2 cells, by RT-qPCR and semi-quantitative PCR (Fig. 1A) and by Western blot (Fig. 1B).

### SENP2 inhibits nuclear translocation of β-catenin by WNT5a in BC cells

**Figure 2:**
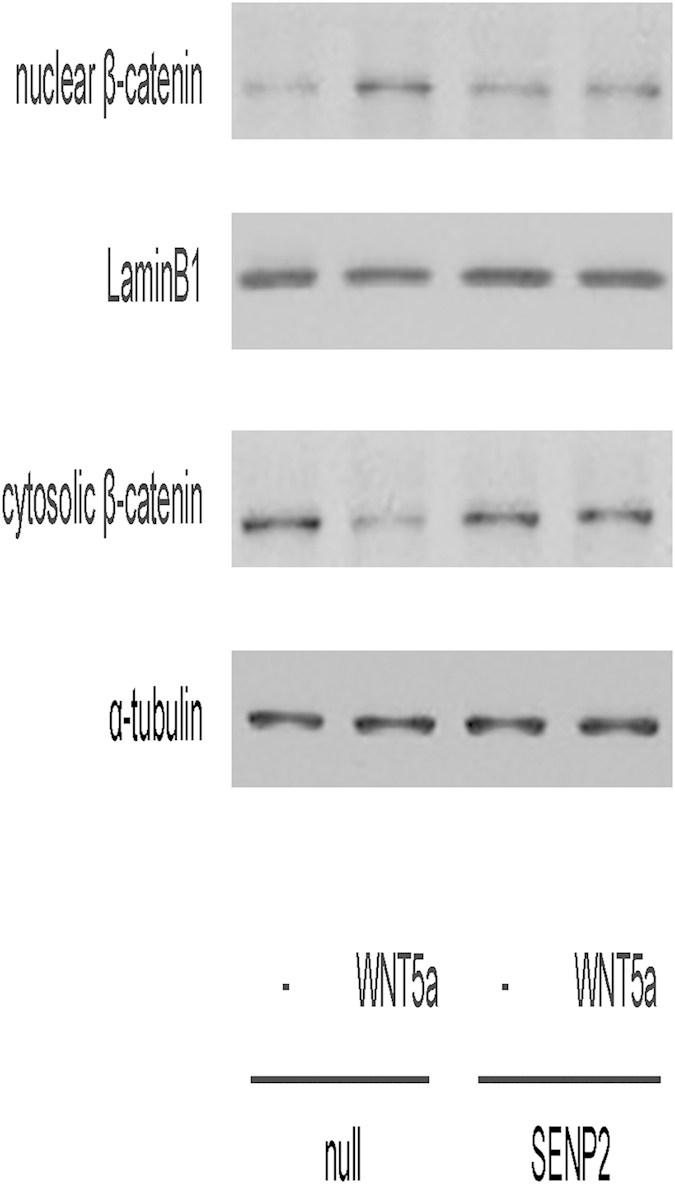
SENP2 inhibits nuclear translocation of β-catenin by WNT5a in BC cells. T24-SENP2 and control T24-null cells were then treated with WNT5a for 30 min and nuclear translocation of β-catenin was analyzed by immunoblotting by analyzed nuclear protein and cytosolic protein separately. The purity of each fraction obtained was validated by immunoblotting for specific markers: for cytoplasmic fractions, we used α-tubulin as a marker, and for nuclear fractions we used LaminB1. N = 5.

T24-SENP2 and control T24-null cells were then treated with WNT5a for 30 min and nuclear translocation of β-catenin was analyzed by immunoblotting. We detected a band with increased density with β-catenin antibody in the nuclear fraction and a band with decreased density with β-catenin antibody in the cytosolic fraction after WNT5a treatment in T24-null cells, suggesting that WNT5a induces nuclear translocation of β-catenin (Fig. 2). However, neither the band density in the nuclear fraction, nor the band density in the cytosolic fraction, was altered in T24-SENP2 cells with β-catenin antibody after WNT5a treatment, suggesting SENP2 may inhibit SUMOylation of some proteins that are required for nuclear translocation of β-catenin by WNT5a in BC cells (Fig. 2). The purity of each fraction obtained was validated by immunoblotting for specific markers: for cytoplasmic fractions, we used α-tubulin as a marker, and for nuclear fractions we used LaminB1 (Fig. 2).

### Nuclear β-catenin binds to the MMP13 promoter

**Figure 3:**
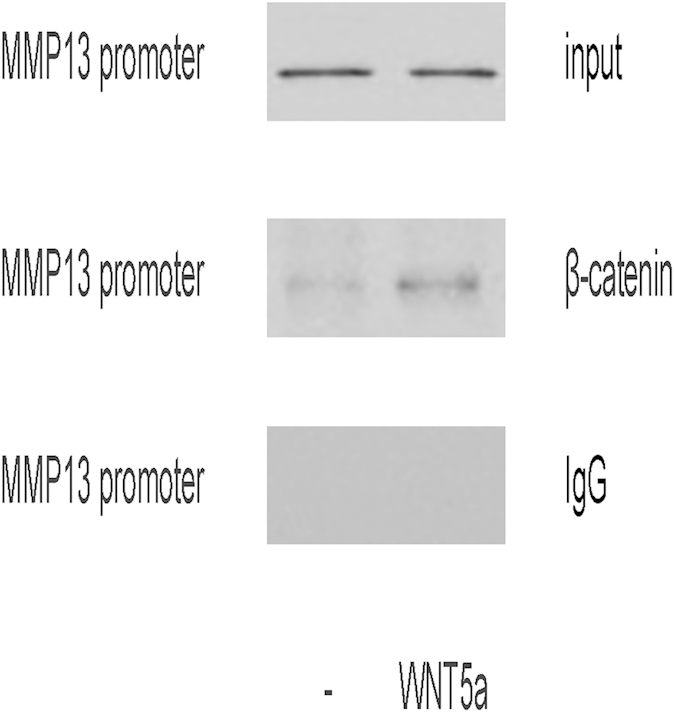
Nuclear β-catenin binds to the MMP13 promoter. We examined whether nuclear β-catenin binds to chromatin that contains the MMP13 promoter region. We immunoprecipitated endogenous nuclear β-catenin from T-24 cells treated with or without WNT5a and amplified the co-purified DNA using PCR. The ChIP isolates contained the promoter region of MMP13, as detected by primers used in semi-quantitative RT-PCR. N = 5.

We next examined whether nuclear β-catenin binds to chromatin that contains the MMP13 promoter region. Thus, we immunoprecipitated endogenous nuclear β-catenin from T-24 cells treated with or without WNT5a and amplified the co-purified DNA using PCR. The ChIP isolates contained the promoter region of MMP13, as detected by primers used in semi-quantitative RT-PCR (Fig. 3). Taken together, these data suggest that endogenous nuclear β-catenin associates with chromatin containing MMP13 promoter, and nuclear β-catenin translocation may activate MMP13 to enhance cancer cell invasiveness in BC cells.

### SENP2 inhibits SUMOylation of TBL1 and TBLR1 by WNT5a in BC cells

**Figure 4:**
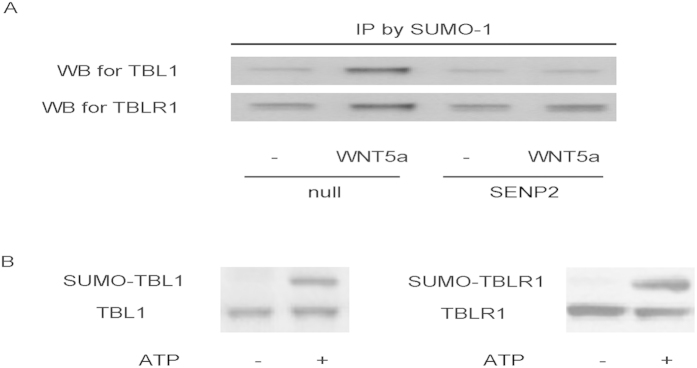
SENP2 inhibits SUMOylation of TBL1 and TBLR1 by WNT5a in BC cells. (**A**) T24-SENP2 and control T24-null cells were treated with WNT5a for 30 min and total cell extracts (200 μg of protein) were immunoprecipitated with anti-SUMO-1 antibody followed by immunoblotting with anti-TBL1 or anti-TBLR1. (**B**) We performed an *in vitro* SUMOylation assay. Slow moving immunoreactive bands of both TBL1 and TBLR1 were recognized by the anti-SUMO antibody. When the assay was performed without ATP as a control, no SUMOylation was detected. N = 5.

Then we studied the molecular mechanisms underlying the WNT5a-induced nuclear translocation of β-catenin that is inhibited by SENP2-mediated SUMOylation suppression. Based on literature, we hypothesize that the WNT signaling-associated proteins TBL1/TBLR1 may be the targets of SUMOylation, since their SUMOylated complex has been shown to enhance nuclear translocation of β-catenin. Thus, T24-SENP2 and control T24-null cells were treated with WNT5a for 30 min and total cell extracts (200 μg of protein) were immunoprecipitated with anti-SUMO-1 antibody followed by immunoblotting with anti-TBL1 or anti-TBLR1. Significant increases in immunoreactive bands for both TBL1 and TBLR1 were seen in the extracts after a 30-min stimulation with WNT5a in T24-null cells, suggesting that both TBL1 and TBLR1 undergo SUMOylation by WNT5a (Fig. 4A). However, in T24-SENP2 cells, no changes in the immunoreactive bands were detected in TBL1 and TBLR1 (Fig. 4A).

To further demonstrate that TBL1 and TBLR1 undergoes SUMOylation, we performed an *in vitro* SUMOylation assay. Slow moving immunoreactive bands of both TBL1 and TBLR1 were recognized by the anti-SUMO antibody (Fig. 4B). When the assay was performed without ATP as a control, no SUMOylation was detected (Fig. 4B), suggesting that SUMOylation of both TBL1 and TBLR1 *in vitro* is not a random event, but a specific process dependent on ATP, which is required in the first step of the SUMOylation process for the activation of SUMO proteins by the E1 heterodimer AOS1-UBA2 enzyme complex. Taken together, these *in vivo* and *in vitro* data suggest that both TBL1 and TBLR1 undergo SUMOylation after WNT5a stimulation.

### SUMOylation of TBL1 and TBLR1 is required for WNT5a-induced β-catenin nuclear translocation in BC cells

Since SUMOylated TBL1/TBLR1 complex has been shown to enhance nuclear translocation of β-catenin, we examined whether they may be responsible for WNT5a-induced β-catenin nuclear translocation in BC cells in this model.

**Figure 5:**
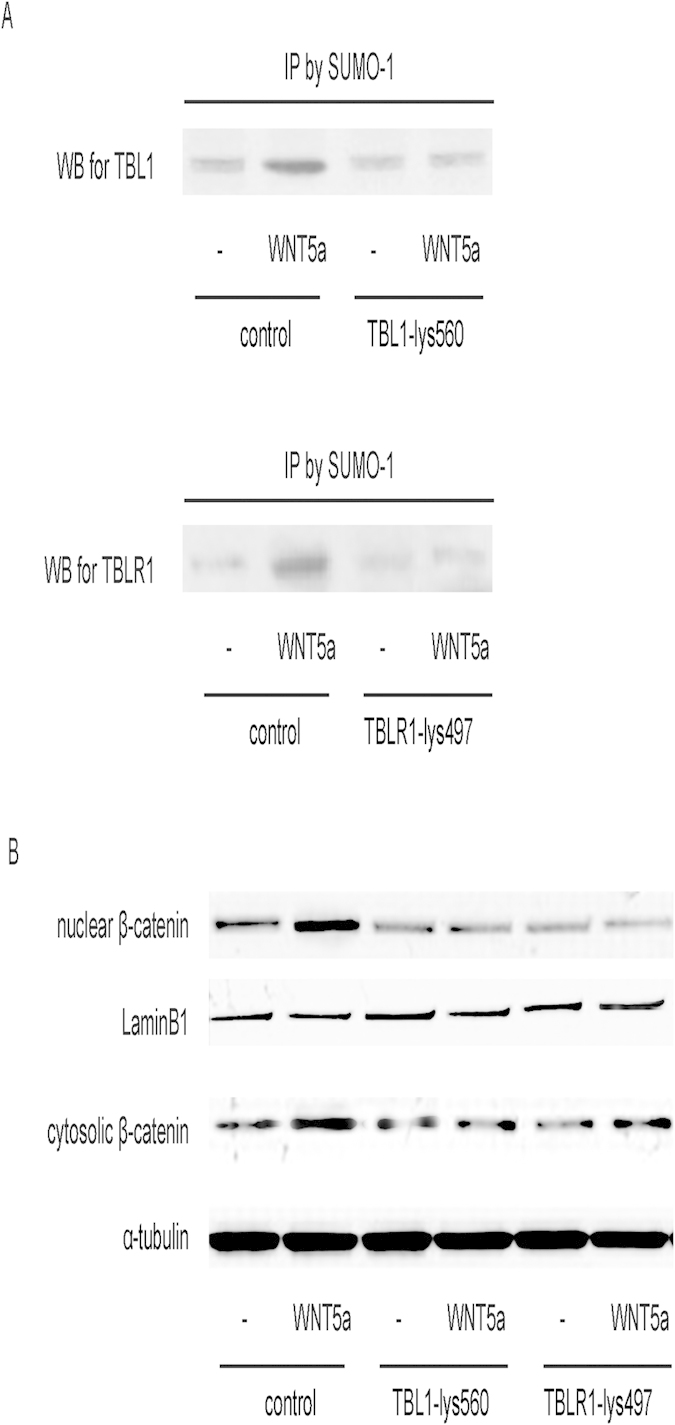
SUMOylation of TBL1 and TBLR1 is required for WNT5a-induced β-catenin nuclear translocation in BC cells. (**A**) We mutated two lysine residues in TBL1 and TBLR1 respectively to arginine residues (TBL1-lys560, TBLR1-lys497). The inhibition of WNT5a-mediated SUMOylation in these mutants was confirmed. (**B**) The nuclear translocation of β-catenin in responsive to WNT5a stimulation in T24 cells that were nucleofected with either mutant was completely abolished. N = 5.

**Figure 6:**
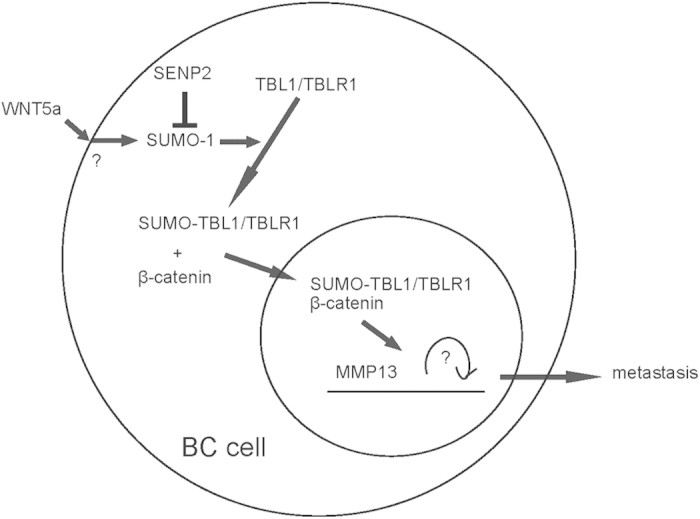
Schematic of the model. SUMOylation of both lysine residues in TBL1 and TBLR1 is required for translocation of β-catenin into the nucleus upon WNT5a stimulation. Hence, SUMOylation of TBL1 and TBLR1 is required for WNT5a-induced β-catenin nuclear translocation in BC cells.

Analyses of the amino acid sequence of TBL1 and TBLR1 by PSORT II software^30^ together with SUMOplot analysis show that one lysine residue in TBL1 (Lys560) and one lysine residue in TBLR1 (Lys497) could be the targets for SUMOylation. Thus, we mutated these two lysine residues in TBL1 and TBLR1 respectively to arginine residues (TBL1-lys560, TBLR1-lys497). The nuclear translocation of β-catenin in responsive to WNT5a stimulation in T24 cells that were nucleofected with either mutant was completely abolished (Fig. 5). These results suggest that SUMOylation of both lysine residues in TBL1 and TBLR1 is required for translocation of β-catenin into the nucleus upon WNT5a stimulation. Hence, SUMOylation of TBL1 and TBLR1 is required for WNT5a-induced β-catenin nuclear translocation in BC cells (Fig. 6).

## Discussion

Previous studies have suggested a link between intracellular the TBL1/TBLR1 protein SUMOylation and the nuclear translocation of β-catenin. However, whether this regulation also exists in BC cells in responsive to WNT ligands as well as the exact underlying molecular mechanisms has not been studied. Here, we show that TBL1/TBLR1 are SUMOylated by WNT5a, which are necessary for WNT5a-mediated β-catenin translocation into the nucleus in human BC cells. More importantly, we found that nuclear β-catenin associated with a chromatin region that corresponds to the promoter region of the human MMP13 gene, suggesting that nuclear β-catenin plays a critical role in the regulation of MMP13 expression at the transcriptional level.

Both TBL1 and TBLR1 were initially identified as core components of the NCoR/SMRT corepressor complexes. The function of TBL1/TBLR1 is dependent on HDAC3. Moreover, TBL1-TBLR1 is also required for the forward-feed repression mediated by NCoR/HDAC3 corepressor complexes via binding of hypoacetylated histones. Thus, SUMOylation of TBL1-TBLR1 enhances binding of TBL1/TBLR1 to β-catenin which subsequently increases in β-catenin-mediated Wnt activation. Analysis of the TBL1/TBLR1 primary amino acid sequence revealed two possible lysine residues (Lys560 on TBL1 and Lys497 on TBLR1) that can accept SUMO moieties. Mutating these residues or overexpression of SENP2, an effective SUMOylation inhibitor, not only inhibited WNT5a-mediated SUMOylation of corresponding protein (TBL1 or TBLR1), but also blocked the nuclear translocation of β-catenin.

We previously reported that overexpression of SENP2 in BC cells inhibited production of MMP13. Here we further show that that disruption of SUMOylation of TBL1/TBLR1 inhibited nuclear translocation of β-catenin, which is necessary for WNT5a-stimulated MMP13 activation. Our ChIP data show that nuclear β-catenin is associated with the region of the MMP13 promoter. Although our studies indicate that β-catenin may interact with the MMP13 promoter, we speculate that this interaction may be direct or indirect. β-catenin does have a DNA binding motif in their primary structure. However, they may also play a role in transcriptional regulation of genes by interacting with certain transcription factors, which contain the basic helix-loop-helix domain to direct bind MMP13 promoter. Further studies are needed to clarify the details.

Together, our data suggest that SUMOylation of both lysine residues in TBL1 and TBLR1 is required for translocation of β-catenin into the nucleus upon WNT5a stimulation. Hence, SUMOylation of TBL1 and TBLR1 is required for WNT5a-induced β-catenin nuclear translocation in BC cells.

## Additional Information

**How to cite this article**: Tan, M. *et al.* SENP2 regulates MMP13 expression in a bladder cancer cell line through SUMOylation of TBL1/TBLR1. *Sci. Rep.* **5**, 13996; doi: 10.1038/srep13996 (2015).
